# Brazilian obligatory subterranean fauna and threats to the hypogean environment

**DOI:** 10.3897/zookeys.746.15140

**Published:** 2018-03-26

**Authors:** Jonas Eduardo Gallão, Maria Elina Bichuette

**Affiliations:** 1 Laboratório de Estudos Subterrâneos, Departamento de Ecologia e Biologia Evolutiva, Universidade Federal de São Carlos, São Carlos, São Paulo, Brasil

**Keywords:** caves, Neotropical region, IUCN Red list, troglobites

## Abstract

The subterranean environment harbors species that are not capable of establishing populations in the epigean environment, i.e., the obligatory subterranean species. These organisms live in a unique selective regime in permanent darkness and usually low food availability, high air humidity in terrestrial habitats, and low temperature range allied to other unique conditions related to lithologies and past climatic influences. The pressure to increase Brazil’s economic growth relies on agricultural/pastoral industries and exporting of raw materials such as iron, limestone, ethanol, soybean, cotton, and meat, as well as huge reservoir constructions to generate electricity. Mining (even on a small scale), agricultural expansion, and hydroelectric projects are extremely harmful to subterranean biodiversity, via the modification and even destruction of hypogean habitats. The Brazilian subterranean species were analyzed with respect to their distributions, presence on the IUCN Red List, and current and potential threats to hypogean habitats. A map and three lists are presented, one with the described obligatory subterranean species, one with undescribed taxa, and one with the current and potential threats to the hypogean environment. To date, 150 obligatory subterranean species have been recorded in Brazil, plus at least 156 undescribed troglomorphic taxa, totaling 306 Brazilian troglobites/obligatory cave fauna. We also analyzed the current and potential cave threats and the conservation actions that are underway to attempt to compensate for loss of these habitats. In according to the Brazilian legislation (Decree 6640) only caves of maximum relevance are fully protected. One strategy to protect the subterranean fauna of Brazil is the inclusion of these species in the IUCN Red List (one of attributes that determines maximum relevance for caves); however, one of the IUCN assumptions is that the taxa must be formally described. It is clear that the description and proposed protection of Brazilian subterranean biodiversity depends on more systematics studies.

## Introduction

The most obvious intrinsic feature of subterranean environments is the absence of light, which results in energy restriction (Poulson and White 1969, Poulson and Lavoie 2000). Furthermore, subterranean environments tend to be environmentally stable in terms of low temperature, high relative humidity, and complete darkness (Moore and Sullivan 1997). Consequently, few organisms are capable of effectively colonizing these environments (Barr 1968).

Obligatory subterranean species have evolved in isolation under particular selective conditions, such as complete darkness, low food quantity (with exceptions), and high and constant air humidity for terrestrial species. Obligatory subterranean species have accumulated specializations that are not present in their epigean relatives, which have culminated in exclusively subterranean populations that are no longer capable of colonizing the epigean realm (Trajano 2012).

The importance and fragility of hypogean environments was acknowledged when subterranean species were placed on the IUCN Red List by the environmental government agency in 2004 (IBAMA) and 2014 (ICMBio) (Machado et al. 2008, ICMBio 444 2014 and ICMBio 445 2014). The inclusion of obligatory subterranean species in the IUCN Red List elevates caves to the maximum relevance level (out of four levels of relevance - maximum, high, median, and low), meaning that the cave habitat must be protected (Decree 6640 from November 7, 2008 (Brasil 2008), Normative Instruction [NI] number 2 from August 20, 2009; Normative Instruction [NI] number 2 from August 30, 2017). The biological attributes present in the Normative Instructions that elevates caves to maximum relevance are species included in official Red Lists; presence of endemic or relict troglobites; presence of rare troglobites; and occurrence of unique ecological interactions.

The hypogean environment is fragile and, thus, highly vulnerable to environmental changes; it typically presents high endemism and small population sizes with low restoration capacity, which implies that obligatory subterranean fauna is sensitive to habitat changes, such as chemical pollution, eutrophication, deforestation close to the outcrops and drainages, uncontrolled tourism, mining, dams, etc. (Poulson 1964, Culver and Pipan 2009).

Extinction rates and disturbances caused by human activities are significant (Pimm et al. 1995), thus the knowledge of biodiversity becomes a fundamental tool to recognize threats to biodiversity. Financial resources for documenting biodiversity must be prioritized, as they are essential to establishing and developing best conservation policies (Brooks et al. 2006).

Knowledge of the geographical distribution of obligatory subterranean fauna in Brazil is fragmented compared to Europe and Asia, where a higher level of knowledge has been achieved (Botosaneanu 1986, Juberthie and Decu 2001, Deharveng et al. 2009, Stoch et al. 2009, Brancelj et al. 2013). The first list of obligatory subterranean fauna of Brazil was published in the 1980s and comprised five areas (Dessen et al. 1980). Since then, these lists have been constantly reviewed (Trajano 1987, Trajano and Gnaspini-Netto 1991, Gnaspini and Trajano 1994, Pinto-da-Rocha 1995, Trajano and Bichuette 2010a). Herein we update and elaborate on the list of Brazilian obligatory subterranean fauna, mapping in detail the areas/regions with this fauna and its main threats.

## Materials and methods

To construct the list, species descriptions, literature data, and sampling conducted by our group were utilized. The undescribed taxa were confirmed by specialists and are deposited in Brazilian collections (Museu Nacional do Rio do Janeiro/Universidade Federal do Rio de Janeiro, Instituto Butantan, Museu de Zoologia da Universidade de São Paulo). The information contained in two existing faunistic lists is expanded upon: one with the formally described obligatory subterranean fauna and the other containing the troglomorphic taxa (possible obligatory subterranean fauna detailed to as accurate taxonomic level as possible).

The purpose of the inclusion of undescribed troglomorphic taxa was to propose potential areas for conservation (since they are not included in the IUCN Red List). To avoid overestimation of taxa, we did not use data from environmental impact assessment reports.

The geomorphologic units used follow Karmann and Sanchez (1979). Groups: main uninterrupted limestone rocks (Una-Irecê, Corumbá, Bambuí, Açungui, Rio Pardo, Araras, Brusque, Apodi); supergroups: main interrupted limestone rocks (Canudos); sandstone: main sandstone rocks (Altamira-Itaituba, Chapada Diamantina); formation: main iron ore rocks (Carajás, Quadrilátero Ferrífero). Since the Bambuí group is huge, we divided it into regions, based on municipalities (Presidente Olegário, Mambaí, São Domingos, São Desidério, Itacarambi, Jaíba, Montes Claros, Cordisburgo, Unaí, Distrito Federal) or based on continuous outcrops (Serra da Canastra, Serra do Ramalho). Other minor geomorphological units used are Serra do Mar and Serra da Mantiqueira (quartiztic), Vargem Alta (marble), and Itirapina (sandstone).

The threats listed herein are those that directly disturb the hypogean environment and its fauna, such as small and large hydroelectrical projects, mining projects, deforestation, uncontrolled tourism, chemical pollution, and lowering of the water table due to extraction of water; and indirect threats such as roads, land conflicts and gas extraction. The main threats were listed for municipalities and for some Brazilian geomorphologic units.

The map was created on QuantumGis Essen 2.14 with shapefiles of South America and Brazil. Besides these, we used the shapefile of Brazilian karst areas, available at the CECAV/ICMBIO website. Circle size is proportional to the number of species occurring in each area and was plotted using Adobe Illustrator CS6.

To evaluate the addition of Brazilian subterranean species in the IUCN Red lists, we compared the number of species presented in the 2004 IUCN Red List (Machado et al. 2008) and the 2014 IUCN Red List (ICMBio 444 2014 and ICMBio 445 2014). We distinguished between the species not rated in the IUCN Red List as “not reported” and “not included”. “Not reported” refers to species that were not revised and “not included” are species that were revised and do not fit into any threat category: vulnerable (VU), endangered (EN), and critically endangered (CR). The term IUCN Red List used herein correspond to the Brazilian List of Threatened Fauna.

## Results

**Figure 1.**
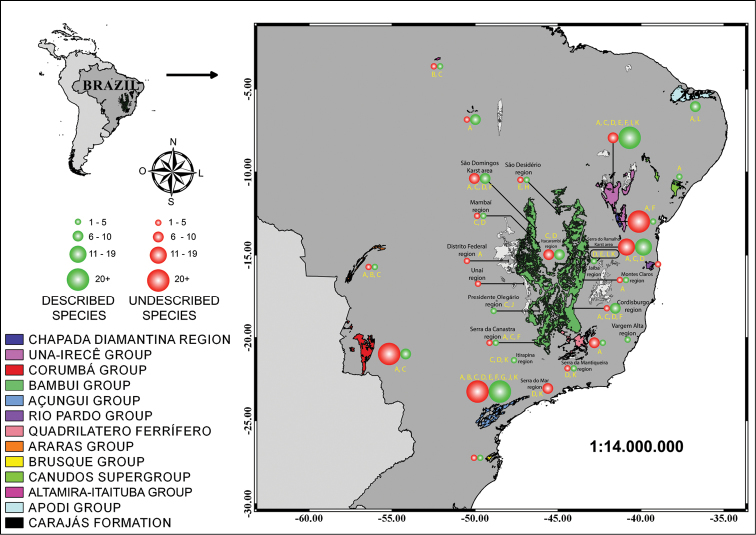
Map of Brazil with main rock groups, karst areas, and formations with obligatory cave-dwelling species. Threats are indicated by letters as follows: **A** Minig **B** Reservoir construction **C** Deforestation for pastures **D** Deforestation for agriculture **E** Pollution of subterranean drainages **F** Tourism **G** Land conflict **H** Road construction, **I** Lowering of water table **J** Small hydroelectric power station buildings, **K** Pesticides **L** Natural gas and oil exploration. For Bambuí group, we grouped as follows (see Table 1 for distinction): Mambaí region - Mambaí and Posse municipalities; Distrito Federal region - Distrito Federal region plus Formosa and Padre Bernardo municipalities; Presidente Olegário region - Presidente Olegário and Vazante municipalities; Serra da Canastra region - São Roque de Minas, Arcos and Pains municipalities; Cordisburgo region - Cordisburgo, Matozinhos, Sete Lagoas, Morro do Pilar, Monjolos and Lagoa Santa municipalities; Montes Claros region - Montes Claros, Coração de Jesus and Luislândia municipalities.

Presently, Brazil has 150 described obligatory subterranean species, distributed over 12 states and located in different lithologies and geomorphologic groups (Figure 1, Table 1). The majority of these species occur in limestone rocks (123 species), mainly owing to the vast size of limestone geomorphologic units and the higher sampling effort in this lithology. Even with the high number of impact reports (mainly mining) regarding iron ore lithologies, and the increase in studies and inventories over the last ten years after publication of Decree 6640, there has been few described species (twelve species). In the other lithologies, sandstone contains less described species than does iron ore (ten species); for quartzitic and marble lithologies, we recorded only two obligatory subterranean species, one for each. Besides, there are two hyporheic fishes, one from Pará State and another from Rondônia State.

**Table 1.** Obligatory subterranean fauna described in Brazil (149 species) and IUCN Red List threatened species categories. VU – vulnerable; EN – endangered; CR – critically endangered; LC – least concern; DD – data deficient. SNR – still not rated, see text for explanations. States: BA – Bahia, GO – Goiás, MG – Minas Gerais, MS – Mato Grosso do Sul, MT – Mato Grosso, PA – Pará, PR – Paraná, RO – Rondônia, RN – Rio Grande do Norte, SP – São Paulo.

Higher taxon	Species	Lithology / Geomorphological Unit / Karstic area or Region (State)	Category 2004	Category 2014
Phylum Platyhelminthes				
Class Turbellaria				
Order Tricladida				
Dimarcusidae	*Hausera hauseri* Leal-Zanchet & Souza, 2014	Limestone / Apodi group / Felipe Guerra region (RN)	–	SNR
Dugesiidae	*Girardia multidiverticulata* Souza, Morais, Cordeiro & Leal-Zancheti, 2015	Limestone / Corumbá group / Serra da Bodoquena karst area (MS)	–	SNR
	*Girardia desiderensis* Souza & Leal-Zancheti, 2016	Limestone / Bambuí group / São Desidério region (BA)	–	SNR
Phylum Porifera				
Class Demospongiae				
Order Haplosclerida				
Spongillidae	**Racekiela cavernicola** Volkmer-Ribeiro, Bichuette & Machado, 2010	Limestone / Una-Irecê group / Morro do Chapéu region (BA)	–	CR
Phylum Arthropoda				
Class Malacostraca				
Order Amphipoda				
Hyalellidae	*Hyalella caeca* Pereira, 1989	Limestone / Açungui group / Alto do Ribeira karst area (SP)	VU	SNR
	*Hyalella spelaea* Bueno & Cardoso, 2011	Sandstone / Itirapina region (SP)	–	SNR
	*Hyalella veredae* Cardoso & Bueno, 2014	Limestone / Bambuí group / Vazante formation / Presidente Olegário region (MG)	–	SNR
	*Hyalella formosa* Cardoso &Araujo, 2014	Limestone / Açungui group / Alto do Ribeira karst area (PR)	–	SNR
	*Hyalella epikarstica* Rodrigues, Bueno & Ferreira, 2014	Limestone / Açungui group / Alto do Ribeira karst area (SP)	–	SNR
Artesiidae	*Megagidiella azul* Koenemann & Holsinger, 1999	Limestone / Corumbá group / Serra da Bodoquena karst area (MS)	“Not reported”	SNR
	*Spelaeogammarus bahiensis* Brum, 1975	Limestone / Una-Irecê group / Curaça region (BA)	“Not reported”	SNR
	*Spelaeogammarus santanensis* Koenemann & Holsinger, 2000	Limestone / Bambuí group / Serra do Ramalho karst area (BA)	“Not reported”	SNR
	*Spelaeogammarus spinilacertus* Koenemann & Holsinger, 2000	Limestone / Una-Irecê group / Iraquara region (BA)	“Not reported”	SNR
	*Spelaeogammarus trajanoae* Koenemann & Holsinger, 2000	Limestone / Una-Irecê group / Campo Formoso region (BA)	“Not reported”	SNR
	*Spelaeogammarus titan* Senna, Andrade, Castelo-Branco & Ferreira, 2014	Limestone / Bambuí group / Serra do Ramalho karst area (BA)	–	SNR
	*Spelaeogammarus sanctus* Bastos-Pereira & Ferreira, 2015	Limestone / Bambuí group / Serra do Ramalho karst area (BA)	–	SNR
	*Spelaeogammarus uai* Bastos-Pereira & Ferreira, 2017	Limestone / Bambuí group / Itacarambi region (MG)	–	–
Mesogammaridae	*Potiberaba porakuara* Fiser, Zagmajter & Ferreira, 2013	Limestone / Apodi group / Felipe Guerra region (RN)	–	SNR
Seborgiidae	*Seborgia potiguar* Fiser, Zagmajter & Ferreira, 2013	Limestone / Apodi group / Governador Dix-Sept Rosado region (RN)	–	SNR
Order Decapoda				
Aeglidae	*Aegla cavernicola* Turkay, 1972	Limestone / Açungui group / Alto do Ribeira karst area (SP)	VU	CR
Aeglidae	*Aegla leptochela* Bond-Buckup & Buckup ,1994	Limestone / Açungui group / Alto do Ribeira karst area (SP)	VU	CR
	*Aegla microphthalma* Bond-Buckup & Buckup, 1994	Limestone / Açungui group / Alto do Ribeira karst area (SP)	VU	CR
Order Isopoda				
Calabozoidae	*Pongycarcinia xiphidiourus* Messana, Baratti & Benvenuti, 2002	Limestone / Una-Irecê group / Campo Formoso region (BA)	“Not reported”	SNR
Brasileirinidae	*Brasileirinho cavaticus* Prevorcnik, Ferreira & Sket, 2011	Limestone / Canudos supergroup / Paripiranga region (BA)	–	SNR
Philosciidae	*Benthana iporangensis* Lima & Serejo, 1993	Limestone / Açungui group / Alto do Ribeira karst area (SP)	“Not reported”	SNR
	*Leonardossia hassalli* Campos-Filho, Araujo & Taiti, 2014	Sandstone / Altamira-Itaituba group / Altamira region (PA)	–	SNR
Pudeoniscidae	*Iansaoniscus iraquara* Campos-Filho, Araujo & Taiti, 2017	Limestone / Una-Irecê group / Iraquara region (BA)	–	–
	*Iansaoniscus georginae* Campos-Filho, Araujo & Taiti, 2017	Limestone / Canudos supergroup / Paripiranga region (BA)	–	–
Scleropactidae	*Amazoniscus eleonorae* Souza, Ferreira & Araujo, 2006	Sandstone / Altamira-Itaituba group / Altamira region (PA)	–	SNR
	*Amazoniscus leistikowi* Campos-Filho, Araujo & Taiti, 2014	Sandstone / Altamira-Itaituba group / Altamira region (PA)	–	SNR
	*Circoniscus buckupi* Campos-Filho & Araujo, 2011	Iron ore / Carajás formation / Parauapebas region (PA)	–	SNR
	*Circoniscus carajasensis* Campos-Filho & Araujo, 2011	Iron ore / Carajás formation / Canãa dos Carajás region (PA)	–	SNR
Styloniscidae	*Spelunconiscus castroi* Campos-Filho, Araujo & Taiti, 2014	Limestone / Bambuí group / Matozinhos region (MG)	–	SNR
	*Xangoniscus aganju* Campos-Filho, Araujo & Taiti, 2014	Limestone / Bambuí group / Serra do Ramalho karst area (BA)	–	SNR
	*Xangoniscus odara* Campos-Filho & Taiti, 2016	Limestone / Bambuí group / Itacarambi region (MG)	–	SNR
	*Iuiuniscus iuiuensis* Souza, Ferreira & Senna, 2015	Limestone / Bambuí group / Serra do Ramalho karst area (BA)	–	SNR
	*Xangoniscus itacarambiensis* Bastos-Pereira, Souza & Ferreira, 2017	Limestone / Bambuí group / Itacarambi region (MG)	–	–
Order Spelaeogriphacea				
Spelaeogriphidae	*Potiicoara brasiliensis* Pires, 1987	Limestone / Corumbá and Araras groups / Serra da Bodoquena karst area (MS) and Rosário Oeste region (MT)	“Not reported”	SNR
Class Chelicerata				
Order Amblypygi				
Charinidae	*Charinus troglobius* Baptista & Giupponi, 2002	Limestone / Bambuí group / Serra do Ramalho karst area (BA)	CR	CR
	*Charinus eleonorae* Baptista & Giupponi, 2003	Limestone / Bambuí group / Itacarambi region (MG)	“Not reported”	CR
	*Charinus caatingae* Vasconcelos & Ferreira, 2016	Limestone / Una-Irecê group / Várzea Nova region (BA)	–	–
	*Charinus taboa* Vasconcelos, Giupponi & Ferreira, 2016	Limestone / Bambuí group / Sete Lagoas region (MG)	–	SNR
	*Charinus ferreus* Giupponi & Miranda, 2016	Iron ore / Carajás formation / Serra de Carajás (PA)	–	SNR
	*Charinus spelaeus* Vasconcelos & Ferreira, 2017	Limeston / Bambuí group / Presidente Juscelino region (MG)	–	–
Order Araneae				
Theraphosidae	*Tmesiphantes hypogeous* Bertani, Bichuette & Pedroso, 2013	Sandstone / Chapada Diamantina region (BA)	–	CR
Dipluridae	*Harmonicon cerberus* Pedroso & Baptista, 2014	Iron ore / Carajás formation / Parauapebas region (PA)	–	CR
Ctenidae	*Isoctenus corymbus* Polotow, Brescovit & Pellegatti-Franco, 2005	Limestone / Bambuí group / São Domingos karst area (GO)	–	CR
Ochyroceratidae	*Speocera eleonorae* Baptista, 2003	Limestone / Corumbá group / Serra da Bodoquena karst area (MS)	“Not reported”	EN
Ochyroceratidae	*Ochyrocera ibitipoca* Baptista, Gonzalez & Tourinho, 2008	Quartzitic / Serra da Mantiqueira / Lima Duarte (MG)	–	EN
Pholcidae	*Metagonia diamantina* Machado, Ferreira & Brescovit, 2011	Limestone / Una-Irecê group / Itaetê region (BA)	–	CR
	*Metagonia potiguar* Ferreira, Souza, Machado & Brescovit, 2011	Limestone / Apodi group / Felipe Guerra region (RN)	–	CR
Prodidomidae	*Lygromma ybyguara* Rheims & Brescovit, 2004	Limestone / Bambuí group / Cordisburgo region (MG)	–	CR
Symphytognathidae	*Anapistula guyri* Rheims & Brescovit, 2003	Limestone / Bambuí group / São Domingos karst area (GO)	VU	LC
Order Opiliones				
Gerdesiidae	*Gonycranaus pluto* Bragagnolo, Hara & Pinto-da-Rocha, 2015	Limestone / Bambuí group / Morro do Pilar region (MG)	–	–
Gonyleptidae	*Pachylospeleus strinatii* Šilhavý, 1974	Limestone / Açungui group / Alto do Ribeira karst area (SP)	VU	EN
	*Iandumoema uai* Pinto-da-Rocha, 1996	Limestone / Bambuí group / Itacarambi region (MG)	CR	CR
	*Iandumoema smeagol* Pinto-da-Rocha, Fonseca-Ferreira & Bichuette, 2015	Limestone / Bambuí group / Monjolos region (MG)	–	–
	*Iandumoema setimapocu* Hara & Pinto-da-Rocha, 2008	Limestone / Bambuí group / Coração de Jesus region (MG)	–	EN
	*Giupponia chagasi* Pérez & Kury, 2002	Limestone / Bambuí group / Serra do Ramalho karst area (BA)	CR	CR
	*Discocyrtus pedrosoi* Kury, 2008	Sandstone / Chapada Diamantina region (BA)	–	LC
	*Eusarcus elinae* Kury, 2008	Limestone / Una-Irecê group / Iraquara region (BA)	–	EN
	*Spinopilar moria* Kury & Pérez-González, 2008	Limestone / Bambuí group / Cordisburgo region (MG)	–	CR
Escadabiidae	*Spaeleoleptes spaeleus* Soares, 1966	Limestone / Bambuí group / Cordisburgo region (MG)	EN	EN
Kimulidae	*Relictopiolus galadriel* Pérez-González, Monte & Bichuette, 2017	Limestone / Bambuí group / Itacarambi region (MG)	–	–
Order Palpigradi				
Eukoeneniidae	*Eukoenenia maquinensis* Souza & Ferreira, 2010	Limestone / Bambuí group / Cordisburgo region (MG)	–	CR
	*Eukoenenia spelunca* Souza & Ferreira, 2011	Marble / Vargem Alta region (ES)	–	CR
	*Eukoenenia virgemdalapa* Souza & Ferreira, 2012	Limestone / Bambuí group / Vazante formation / Vazante region (MG)	–	EN
	*Eukoenenia sagarana* Souza & Ferreira, 2012	Limestone / Bambuí group / Cordisburgo region (MG)	–	CR
	*Eukoenenia jequitinhonha* Souza & Ferreira, 2016	Granitic / Caraí region (MG)	–	–
	*Eukoenenia cavatica* Souza & Ferreira, 2016	Limestone / Bambuí group / Arcos region	–	–
Order Pseudoscorpiones				
Bochicidae	*Spelaeobochica allodentatus* Mahnert, 2001	Limestone / Una-Irecê group / Palmeiras region (BA)	“Not reported”	CR
	*Spelaeobochica muchmorei* Andrade & Mahnert, 2003	Limestone / Açungui group / Alto do Ribeira karst area (SP)	“Not reported”	EN
	*Spelaeobochica iuiu* Ratton, Mahnert & Ferreira, 2012	Limestone / Bambuí group / Serra do Ramalho karst area (BA)	–	CR
Chthoniidae	*Maxchernes iporangae* Mahnert & Andrade, 1998	Limestone / Açungui group / Alto do Ribeira karst area (SP)	EN	CR
	*Pseudochthonius strinatii* Beier, 1969	Limestone / Açungui and Bambuí groups / Alto do Ribeira karst area (SP-PR) and Sete Lagoas region (MG)	VU	DD
	*Pseudochthonius biseriatus* Mahnert, 2001	Limestone / Bambuí group / Itacarambi region (MG)	“Not reported”	CR
Ideoroncidae	*Ideoroncus cavicola* Mahnert 2001	Limestone / Açungui group / Alto do Ribeira karst area / Iporanga (SP) and Rio Branco do Sul regions (PR)	“Not reported”	VU
Order Scorpiones				
Buthidae	*Troglorhopalurus translucidus* Lourenço, Baptista & Giupponi, 2004	Sandstone / Chapada Diamantina region (BA)	–	EN
	*Troglorhopalurus lacrau* (Lourenço & Pinto-da-Rocha, 1997)	Limestone / Una-Irecê group / Itaetê region (BA)	“Not reported”	EN
Class Chilopoda				
Order Scolopendromorpha				
Cryptopidae	Cryptops (Trygonocryptops) iporangensis Ázara & Ferreira, 2013	Limestone / Açungui group / Alto do Ribeira karst area (SP)	–	EN
	Cryptops (Cryptops) spelaeoraptor Ázara & Ferreira, 2014	Limestone / Una-Irecê group / Campo Formoso region (BA)	–	VU
	*Scolopocryptops troglocaudatus* Chagas-Jr. & Bichuette, 2015	Sandstone / Chapada Diamantina region (BA)	–	SNR
Scolopocryptopidae	Newportia (Newportia) spelaea Ázara & Ferreira, 2014	Limestone / Una-Irecê group / Campo Formoso region (BA)	–	–
	Newportia (Newportia) potiguar Ázara & Ferreira, 2014	Limestone / Apodi group / Apodi and Felipe Guerra regions (RN)	–	–
Class Diplopoda				
Order Glomeridesmida				
Glomeridesmidae	*Glomerides musspelaeus* Iniesta & Wesewer, 2012	Iron ore / Carajás formation / Curionópolis region (PA)	–	CR
Order Spirostreptida				
Pseudonannolenidae	*Pseudonannolene spelaea* Iniesta & Ferreira, 2013	Iron ore / Carajás formation / Parauapebas region (PA)	–	CR
	*Pseudonannolene ambuatinga* Iniesta & Ferreira, 2013	Limestone / Bambuí group / Pains region (MG)	–	EN
	*Pseudonannolene lundi* Iniesta & Ferreira, 2015	Limestone / Bambuí group / Luislândia region (MG)	–	SNR
Order Polydesmida				
Chelodesmidae	*Leodesmus yporangae* (Schubart, 1946)	Limestone / Açungui group / Alto do Ribeira karst area (SP)	VU	CR
Cryptodesmidae	*Peridontodesmella alba* Schubart, 1957	Limestone / Açungui group / Alto do Ribeira karst area / Iporanga (SP) and Adrianópolis regions (PR)	VU	EN
Fuhmannodesmidae	*Phaneromerium cavernicolum* Golovatch & Wytwer, 2004	Limestone / Bambuí group / Serra do Ramalho karstarea (BA)	“Not reported”	–
Pyrgodesmidae	*Yporangiella stygius* Schubart ,1946	Limestone / Açungui group / Alto do Ribeira karst area (SP)	VU	VU
Class Entognatha				
Order Diplura				
Campodeidae	*Oncinocampa trajanoae* Condé, 1997	Limestone / Açungui group / Alto do Ribeira karst area (SP)	“Not reported”	SNR
Order Collembola				
Arrhopalitidae	*Arrhopalites amorimi* Palacios-Vargas & Zeppelini, 1995	Limestone / Açungui group / Alto do Ribeira karst area (SP)	VU	CR
	*Arrhopalites gnaspinii* Palacios-Vargas & Zeppelini, 1995	Limestone / Açungui group / Alto do Ribeira karst area (SP)	VU	CR
	*Arrhopalites lawrencei* Palacios-Vargas & Zeppelini, 1995	Limestone / Açungui group / Alto do Ribeira karst area (SP)	VU	CR
	*Arrhopalites alambariensis* Zeppelini, 2006	Limestone / Açungui group / Alto do Ribeira karst area (SP)	–	CR
	*Arrhopalites botuveraensis* Zeppelini, 2006	Limestone / Brusque group / Botuverá region (SC)	–	CR
	*Arrhopalites heteroculatus* Zeppelini, 2006	Limestone / Açungui group / Alto do Ribeira karst area (SP)	–	CR
	*Arrhopalites paranaensis* Zeppelini, 2006	Limestone / Açungui group / Alto do Ribeira karst area (PR)	–	CR
Hypogastruridae	*Acherontides eleonorae* Palacios-Vargas & Gnaspini-Neto, 1992	Limestone / Açungui group / Alto do Ribeira karst area / Iporanga (SP) and Rio Branco do Sul regions (PR)	“Not reported”	EN
Paronellidae	*Troglobius brasiliensis* Palacios-Vargas & Zeppelini, 1995	Sandstone / Altamira-Itaituba region / Medicilândia region (PA); Limestone / Açunguigroup / Alto do Ribeira karst area (SP)	“Not reported”	CR
	*Troglobius ferroicus* Zeppelini, Silva & Palácios-Vargas, 2014	Iron ore / Quadrilátero Ferrífero formation / Itabirito region (MG)	–	CR
	*Trogolaphys aaelleni* Yossi, 1988	Limestone / Açungui group / Alto do Ribeira karst area (SP)	VU	VU
	*Trogolaphysa hauseri* Yossi, 1989	Limestone / Açungui group / Alto do Ribeira karst area (SP)	VU	VU
Sminthuridae	*Pararrhopalites wallacei* (Palacios-Vargas & Zeppelini, 1995)	Limestone / Açungui group / Alto do Ribeira karst area (SP)	VU	CR
	*Pararrhopalites papaveroi* (Zeppelini & Palacios-Vargas, 1999)	Limestone / Corumbá group / Serra da Bodoquena karst area (MS)	VU	EN
Class Insecta				
Order Zygentoma				
Nicoletiidae	*Cubacubana spelaea* Galán, 2001	Limestone / Una-Irecê group / Campo Formoso region (BA)	“Not reported”	SNR
Order Blattaria				
Blattellidae	*Litoblatta camargoi* Gutierrez, 2005	Limestone / Una-Irecê group / Iraquara region (BA)	–	SNR
Order Coleoptera				
Carabidae	*Schizogenius ocellatus* Whitehead, 1972	Limestone / Açungui group / Alto do Ribeira karst area (SP)	VU	EN
	*Coarazuphium tessai* (Godoy & Vanin, 1990)	Limestone / Bambuí group / Serra do Ramalho karst area (BA)	VU	CR
	*Coarazuphium bezerra* Gnaspini, Vanin & Godoy, 1998	Limestone / Bambuí group / São Domingos karst area (GO)	VU	VU
	*Coarazuphium cessaima* Gnaspini, Vanin & Godoy, 1998	Limestone / Una-Irecê group / Itaetê region (BA)	VU	CR
	*Coarazuphium pains* Alvares & Ferreira, 2002	Limestone / Bambuí group / Pains region (MG)	VU	EN
	*Coarazuphium formoso* Pellegrini & Ferreira, 2011	Limestone / Una-Irecê group / Campo Formoso region (BA)	–	VU
	*Coarazuphium tapiaguassu* Pellegrini & Ferreira, 2011	Iron ore / Carajás formation / Curionópolis region (PA)	–	CR
	*Coarazuphium caatinga* Pellegrini & Ferreira, 2014	Limestone / Una-Irecê group / Campo Formoso region (BA)	–	EN
	*Coarazuphium ricardoi* Bená & Vanin, 2014	Limestone / Açungui group / Alto do Ribeira karst area (PR)	–	CR
	*Coarazuphium spinifemur* Pellegrini & Ferreira, 2017	Iron ore / Carajás formation / Curionópolis region (PA)	–	–
	*Coarazuphium amazonicus* Pellegrini & Ferreira, 2017	Iron ore / Carajás formation / Flona de Carajás (PA)	–	–
Dytiscidae	*Copelatus cessaima* Caetano, Bená & Vanin, 2013	Iron ore / Carajás formation / Parauapebas region (PA)	–	CR
Staphylinidae	*Metopiellus painensis* Asenjo, Ferreira & Zampaulo, 2017	Limestone / Bambuí group / Pains region (MG)	–	–
Order Hemiptera				
Cixiidae	*Ferricixius davidi* Hoch & Ferreira, 2012	Iron ore / Quadrilátero Ferrífero formation / Itabirito region (MG)	–	SNR
Kinnaridae	*Kinnapotiguara troglobia* (Hoch & Ferreira, 2013)	Limestone / Apodi group / Felipe Guerra and Governador Dix-Sept Rosado regions (RN)	–	SNR
	*Iuiuia caeca* Hoch & Ferreira, 2016	Limestone / Bambuí group / Serra do Ramalho karst area (BA)	–	SNR
Order Orthoptera				
Phalangopsidae	*Endecous apterus* Bolfarini & Souza-Dias, 2013	Limestone / Una-Irecê group / Ituaçu region (BA)	–	SNR
	*Endecous peruassuensis* Bolfarini, 2015	Limestone / Bambuí group / Itacarambi region (MG)	–	–
Phylum Mollusca				
Class Gastropoda				
Order Mesogastropoda				
Hydrobiidae	*Potamolithus troglobius* Simone & Moracchiolli, 1999	Limestone / Açungui group / Alto do Ribeira karst area (SP)	VU	CR
Order Caenogastropoda				
Pomatiopsidae	*Spiripockia punctata* Simone, 2012	Limestone / Bambuí group / Serra do Ramalho karst area (BA)	–	EN
Phylum Chordata				
Class Osteichtyes				
Order Characiformes				
Characidae	*Stygichthys typhlops* Brittan & Böhlke, 1965	Limestone / Bambuí group / Jaíba region (MG)	VU	EN
Order Gymnotiformes				
Sternopygidae	*Eigenmannia vicentespelaea* Triques, 1996	Limestone / Bambuí group / São Domingos karst area (GO)	VU	VU
Order Siluriformes				
Callychthyidae	*Aspidoras mephisto* Tencatt & Bichuette, 2017	Limestone / Bambuí group / Posse region (GO)	“Not reported”	EN
Heptapteridae	*Pimelodella kronei* (Ribeiro, 1907)	Limestone / Açungui group / Alto do Ribeira karst area (SP)	VU	EN
	*Pimelodella spelaea* Trajano, Reis & Bichuette, 2004	Limestone / Bambuí group / São Domingos karst area (GO)	–	EN
	*Rhamdia enfurnada* Bichuette & Trajano, 2005	Limestone / Bambuí group / Serra do Ramalho karst area (BA)	–	LC
	*Rhamdiopsis krugi* Bockmann & Castro, 2010	Limestone / Una-Irecê group / Itaetê region (BA)	–	VU
Loricariidae	*Ancistrus cryptophthalmus* Reis, 1987	Limestone / Bambuí group / São Domingos karst area (GO)	“Not reported”	EN
	*Ancistrus formoso* Sabino & Trajano, 1997	Limestone / Corumbá group / Serra da Bodoquena karst area (MS)	VU	VU
Trichomycteridae	*Trichomycterus itacarambiensis* Trajano & de Pinna, 1996	Limestone / Bambuí group / Itacarambi region (MG)	VU	CR
	*Trichomycterus dali* Rizzato, Costa-Jr, Trajano & Bichuette, 2011	Limestone / Corumbá group / Serra da Bodoquena karst area (MS)	–	VU
	*Trichomycterus rubbioli* Bichuette & Rizzato, 2012	Limestone / Bambuí group / Serra do Ramalho karst area (BA)	–	VU
	*Ituglanis mambai* Bichuette & Trajano, 2008	Limestone / Bambuí group / Posse region (GO)	–	EN
	*Ituglanis bambui* Bichuette & Trajano, 2004	Limestone / Bambuí group / São Domingos karst area (GO)	–	CR
	*Ituglanis passensis* Fernandez & Bichuette, 2002	Limestone / Bambuí group / São Domingos karst area (GO)	“Not reported”	VU
	*Ituglanis epikarsticus* Bichuette & Trajano, 2004	Limestone / Bambuí group / São Domingos karst area (GO)	–	VU
	*Ituglanis ramiroi* Bichuette & Trajano, 2004	Limestone / Bambuí group / São Domingos karst area (GO)	–	VU
	*Ituglanis boticário* Rizzato & Bichuette, 2014	Limestone / Bambuí group / Mambaí region (GO)	–	SNR
	*Glaphyropoma spinosum* Bichuette, de Pinna & Trajano, 2008	Sandstone / Chapada Diamantina region (BA)	–	VU
*Incertae sedis*	*Phreatobius cisternarum* Goeldi, 1905	Hyporheic / Ilha de Marajó (PA)	–	“Not reported”
	*Phreatobius dracunculus* Shibatta, Muriel-Cunha & de Pinna, 2007	Hyporheic / Rio Pardo basin (RO)	–	“Not reported”

At least 156 troglomorphic/stygomorphic taxa are undescribed (Figure 1, Table 2), representing possible obligatory subterranean populations; these collections, deposited in different museums, await further taxonomic studies. Most of these specimens have not been identified to even a generic taxonomic level. In total, we listed approximately 306 obligatory and potentially obligatory subterranean species for Brazil (Tables 1 and 2). The Brazilian states with the highest number of species are Bahia (Serra do Ramalho karst area and São Desidério region, part of the Bambuí group, the Una-Irecê and Rio Pardo groups, the Canudos supergroup and the sandstone Chapada Diamantina; at least 90 obligatory subterranean species) and São Paulo (including part of the Açungui group, with at least 66 obligatory subterranean species) (Figure 1). Considering the geomorphological units used here, the Bambui group is the richest with 100 obligatory subterranean species followed by the Açungui group with 73 obligatory subterranean species.

**Table 2.** Obligatory subterranean undescribed. References: A – Dessen et al. 1980; B – Chaimowicz 1984; C – Trajano 1987; D – Trajano and Gnaspini-Netto 1991; E – Trajano and Moreira 1991; F – Gnaspini and Trajano 1994; G – Trajano and Sanchez 1994; H – Pinto-da-Rocha 1995; I – Bichuette 1998; J – Lourenço et al. 2004; K – Deharveng 2005; L – Trajano and Bichuette 2010a; M – Cordeiro et al. 2014; TS – this study. spp – widespread taxa possibly meaning several species. States: BA-Bahia, GO-Goiás, MG-Minas Gerais, MS-Mato Grosso do Sul, MT-Mato Grosso, PA-Pará, PR-Paraná, RJ-Rio de Janeiro, RN-Rio Grande do Norte, SC-Santa Catarina, SP-São Paulo.

Taxon	Lithology / Geomorphological Unit / karst area or region	References
Phylum Annelida		
Class Clitellata		
Subclass Oligochaeta	Limestone / Corumbá group / Serra da Bodoquena karst area (MS)	M
Phylum Platyhelminthes		
Order Tricladida		
Dugesiidae indet. 1	Limestone / Bambuí group / Serra do Ramalho karst area (BA)	TS
Dugesiidae indet. 2	Limestone / Açungui group / Alto do Ribeira karst area (SP)	L
Phylum Onychophora		
Order Euonychophora		
Peripatidae indet.	Limestone / Corumbá group / Serra da Bodoquena karst area (MS)	M
Phylum Arthropoda		
Order Amphipoda		
Bogidiellidae		
*Megagidiella* sp.	Limestone / Corumbá group / Serra da Bodoquena karst area (MS)	L
Hyalellidae		
Hyalella aff. pernix	Limestone / Açungui group / Alto do Ribeira karst area (SP)	F, G, H
*Hyalella* sp.	Limestone / Açungui group / Alto do Ribeira karst area (SP)	A, D, G, H
Order Isopoda		
Indet. 1	Limestone / Bambuí group / Montes Claros region (MG)	A, B, H
Indet. 2	Limestone / Araras group / Nobres region (MT)	L
So. Oniscidea	Limestone / Corumbá group / Serra da Bodoquena karst area (MS)	M
Armadillidae		
*Venezillo* sp. 1	Magnesita / Padre Bernardo region (GO)	F, H
*Venezillo* sp. 2	Limestone / Bambuí group / Distrito Federal region (GO)	L
Bathytropidae		
*Neotroponiscus* sp.	Iron ore / Quadrilátero Ferrífero formation / Brumadinho region (MG)	Cardoso & Araujo pers. comm.
Philosciidae indet. 1	Limestone / Açungui group / Alto do Ribeira karst area (SP)	F, H
Philosciidae indet. 2	Sandstone / Chapada Diamantina region (BA)	TS
*Benthana* sp.	Limestone / Açungui group / Alto do Ribeira karst area (SP)	F, G, H
Platyarthridae		
*Trichorhina* spp.	Limestone / Bambuí group / several regions (BA, MG, SP, PR); Iron ore / Quadrilátero Ferrífero (MG)	H, L
Scleropactidae indet.	Sandstone / Altamira-Itaituba group / Altamira region (PA)	E, F, G, H, L
Styloniscidae indet. 1	Limestone / Bambuí group / Itacarambi region (MG)	TS
Styloniscidae indet. 2	Limestone / Bambuí group / Itacarambi region and Serra do Ramalho karst area (MG and BA)	L, TS
Styloniscidae indet. 3	Limestone / Bambuí group / Serra do Ramalho karst area (BA)	TS
Styloniscidae indet. 4	Limestone / Bambuí group / Serra do Ramalho karst area (BA)	TS
Styloniscidae indet. 5	Limestone / Bambuí group / Serra do Ramalho karst area (BA)	F, G, H
Styloniscidae indet. 6	Limestone / Açungui group / Alto do Ribeira karst area (SP)	F, H
Styloniscidae indet. 7	Limestone / Açungui group / Alto do Ribeira karst area (SP)	F, H
Styloniscidae indet. 8	Limestone / Bambuí group / Serra do Ramalho karst area (BA)	B, G, H
Styloniscidae indet. 9	Sandstone / Chapada Diamantina region (BA)	TS
*Pectenoniscus* sp. 1	Limestone / Brusque group / Botuverá region (SC)	L
*Pectenoniscus* sp. 2	Limestone / Bambuí group / Serra do Ramalho karst area (BA)	TS
*Pectenoniscus* sp. 3	Limestone / Bambuí group / Lagoa Santa region (MG)	L
*Pectenoniscus* sp. 4	Limestone / Açungui group / Alto do Ribeira karst area (SP)	H, L
Order Decapoda		
Palaeomonidae		
*Macrobrachium* indet.	Sandstone / Altamira-Itaituba group / Prainha region (PA)	E, G, H
Subclass Acari indet. 1	Sandstone / Chapada Diamantina region (BA)	TS
Subclass Acari indet. 2	Sandstone / Chapada Diamantina region (BA)	TS
Order Amblypygi		
Charinidae		
*Charinus* sp.	Limestone / Bambuí group / Serra do Ramalho karst area (BA)	L, TS
Order Araneae		
Symphytognathidae		
*Anapistula* sp.	Limestone / Açungui group / Alto do Ribeira karst area (SP)	F, G, H
Hahniidae indet.	Limestone / Açungui group / Alto do Ribeira karst area (SP)	E, F, H, L
Amaurobiidae indet.	Limestone / Corumbá group / Serra da Bodoquena karst area (MS)	L
Ctenidae indet.	Limestone / Corumbá group / Serra da Bodoquena karst area (MS)	M
*Isoctenus* sp.	Sandstone / Chapada Diamantina region (BA)	J, L
*Enoploctenus* sp.	Sandstone / Chapada Diamantina region (BA)	TS
Gnaphosidae indet.	Quartzitic / Serra da Mantiqueira / Ibitipoca region (MG)	L
Nesticidae		
*Nesticus* sp. 1	Limestone / Una-Irecê group / Chapada Diamantina region (BA)	L
*Nesticus* sp. 2	Limestone / Bambuí group / Lagoa Santa region (MG)	L
Ochyroceratidae indet. 1	Sandstone / Chapada Diamantina region (BA)	TS
Ochyroceratidae indet. 2	Sandstone / Chapada Diamantina region (BA)	J, L
Ochyroceratidae indet. 3	Limestone / Bambuí group / Serra do Ramalho karst area (BA)	L, TS
*Ochyrocera* sp. 1	Limestone / Bambuí group / São Domingos karst area (GO)	L
*Ochyrocera* sp. 2	Granitic / Serra do Mar / Rio de Janeiro region (RJ)	L
Prodidomidae indet.	Sandstone / Chapada Diamantina region (BA)	TS
cf. Prodidomidae indet.	Limestone / Bambuí group / Serra da Canastra region (MG)	TS
Order Opiliones		
Gonyleptidae indet. 1	Limestone / Brusque group / Botuverá region (SC)	L
Gonyleptidae indet. 2	Limestone / Bambuí group / Serra do Ramalho karst area (BA)	TS
Gonyleptidae indet. 3	Limestone / Açungui group / Alto do Ribeira karst area (SP)	L
Gonyleptidae indet. 4	Sandstone / Chapada Diamantina region (BA)	J
*Eusarcus* sp. 1	Limestone / Corumbá group / Serra da Bodoquena karst area (MS)	L, M
*Eusarcus* sp. 2	Limestone / Bambuí group / São Domingos karst area (GO)	L
*Eusarcus* sp. 3	Sandstone / Chapada Diamantina region (BA)	TS
*Eusarcus* sp. 4	Limestone / Bambuí group / Serra da Canastra region (MG)	TS
Escadabiidae indet	Limestone / Bambuí group / Itacarambi region (MG)	TS
*Spaeleoleptes* sp.	Limestone / Una-Irecê group / Chapada Diamantina region (BA)	L
Order Palpigradi spp.	Limestone / Açunguiand Bambuí groups / Alto do Ribeira karst area and Mambai region (SP and GO)	L
*Eukoenenia* sp.	Sandstone / Chapada Diamantina region (BA)	TS
*Eukoenenia* sp.	Limestone / Araras group / Nobres region (MT)	TS
Order Pseudoscorpiones		
Chernetidae indet.	Sandstone / Chapada Diamantina region (BA)	TS
Chthoniidae indet. 1	Limestone / Açungui group / Alto do Ribeira karst area (SP)	F, H
Chthoniidae indet. 2	Sandstone / Chapada Diamantina region (BA)	TS
Chthoniidae indet. 3	Iron ore / Quadrilátero Ferrífero (MG)	L
Class Diplopoda indet. 1	Limestone / Una-Irecê group / Chapada Diamantina region (BA)	F, H
Diplopoda indet. 2	Limestone / Bambuí group / Unaí region (MG)	D, H
Order Polydesmida indet. 1	Limestone / Bambuí group / Formosa region (GO)	F, G, H
Polydesmida indet. 2	Limestone / Açungui group / Alto do Ribeira karst area (SP)	F, H
Polydesmida indet. 3	Iron ore / Quadrilátero Ferrífero (MG)	L
Polydesmida indet. 4	Limestone / Bambuí group / Itacarambi region (MG)	K, TS
Chelodesmidae indet.	Limestone / Açungui group / Alto do Ribeira karst area (SP)	F, H
*Alecodesmus* sp.	Limestone / Açungui group / Alto do Ribeira karst area (SP)	F, H
Cryptodesmidae indet.	Limestone / Açungui group / Alto do Ribeira karst area (SP)	D, F, G, H
*Cryptodesmus* indet.	Limestone / Açungui group / Adrianópolis region (PR)	H
*Cryptodesmus* sp. 1	Limestone / Açungui group / Alto do Ribeira karst area (SP)	L
*Cryptodesmus* sp. 2	Limestone / Açungui group / Alto do Ribeira karst area (SP)	L
cf. Cryptodesmidae indet.	Limestone / Una-Irecê group / Chapada Diamantina region (BA)	F, H
Oniscodesmidae indet. 1	Limestone / Una-Irecê group / Chapada Diamantina region (BA)	F, H
Oniscodesmidae indet. 2	Granitic / Serra do Mar / Ribeirão Pires region (SP)	F, H
Oniscodesmidae indet. 3	Limestone / Açungui group / Alto do Ribeira karst area (SP)	F, H
*Crypturodesmus* sp. 1	Limestone / Corumbá group / Serra da Bodoquena karst area (MS)	L, M
*Crypturodesmus* sp. 2	Limestone / Açungui group / Alto do Ribeira karst area (SP)	L
*Crypturodesmus* sp. 3	Limestone / Brusque group / Botuverá region (SC)	L
*Katandodesmus* spp.	Limestone / Açungui group / several regions (PR and SP)	F, G, H
*Katandodesmus* sp.	Limestone / Corumbá group / Serra da Bodoquena karst area (MS)	F, G, H, M
Paradoxosomatidae indet.	Limestone / Corumbá group / Serra da Bodoquena karst area (MS)	M
Pyrgodesmidae indet.	Limestone / Una-Irecê group / Chapada Diamantina region (BA)	TS
Order Spirostreptida		
Pseudonannolenidae indet.	Sandstone / Chapada Diamantina region (BA)	TS
Class Chilopoda		
Order Geophilomorpha		
Geophilidae indet.	Limestone / Açungui group / Alto do Ribeira karst area (SP)	L
Order Scolopendromorpha		
Cryptopidae		
*Cryptops* sp.	Iron ore / Carajás Formation / Carajás region (PA)	L
Scolopendridae indet.	Sandstone / Chapada Diamantina region (BA)	TS
Order Lithobiomorpha indet.	Iron ore / Quadrilátero Ferrífero (MG)	L
Class Pauropoda indet.	Sandstone / Altamira-Itaituba group / Altamira region (PA)	TS
Class Symphyla indet.	Limestone / Açungui group / Alto do Ribeira karst area (SP)	L
Scutigerellidae indet.	Limestone / Bambuí group / Serra do Ramalho karst area (BA)	TS
cf. *Hanseniella* sp.	Limestone / Rio Pardo group (BA)	L
Class Entognatha		
Order Collembola indet.	Limestone / Bambuí group / Itacarambi region (MG)	K, TS
Arrhopalitidae indet.	Limestone / Corumbá group / Serra da Bodoquena karst area (MS)	F, G, H
*Arrhopalites* sp.	Iron ore / Quadrilátero Ferrífero (MG)	L
Hypogastruridae		
*Acherontides* spp.	Limestone / Brusque and Rio Pardo groups (SC and BA)	L
Onychiuridae indet.	Limestone / Açungui group / Alto do Ribeira karst area (SP)	F, H
Isotomidae spp.	Granitic, Limestone and Iron ore / Serra do Mar, Bambuí group and Quadrilátero Ferrífero / several regions (SP and MG)	D, F, G, H, L
Entomobryidae spp.	Limestone and Sandstone / Açungui, Bambuí, Corumbá groups and Chapada Diamantina region (BA, GO, MS, PR and SP)	F, G, H, L, M
*Heteromurus* sp.	Sandstone / Chapada Diamantina region (BA)	TS
*Verhoefiella* sp.	Sandstone / Chapada Diamantina region (BA)	TS
Cyphoderidae spp.	Granitic and Limestone / Serra do Mar, Bambuí and Corumbá groups / several regions (BA, GO, MS and SP)	F, G, H, M
*Cyphoderus* sp.	Limestone / Bambuí group / Montes Claros region (MG)	F, H
Paronellidae spp.	Limestone / Açungui, Una-Irecê and Corumbá groups / Alto do Ribeira karst area, Chapada Diamantina region and Serra da Bodoquena karst area (SP, BA and MS)	D, F, H, G
*Trogolaphysa* sp.	Limestone / Corumbá group / Serra da Bodoquena karst area (MS)	M
*Troglopedetes* sp. 1	Sandstone / Chapada Diamantina region (BA)	TS
*Troglopedetes* sp. 2	Limestone / Brusque group / Botuverá region (SC)	L
*Troglobius* sp. 1	Limestone / Açungui group / Alto do Ribeira karst area (SP)	F, H
*Troglobius* sp. 2	Sandstone / Altamira-Itaituba / Prainha region (PA)	E, F, H
Class Insecta		
Order Blattaria		
Blattellidae indet.	Sandstone / Chapada Diamantina region (BA)	TS
Order Coleoptera		
Carabidae indet.	Limestone / Bambuí group / Serra do Ramalho karst area (BA)	TS
*Oxydrepanus* sp.	Limestone / Açungui group / Alto do Ribeira karst area (SP)	F, G, H, L
Dytiscidae indet.	Sandstone / Chapada Diamantina region (BA)	TS
Staphylinidae		
Pselaphinae indet. 1	Limestone / Açungui group / Alto do Ribeira karst area (SP)	C, F, H
Pselaphinae indet. 2	Limestone / Bambuí group / São Domingos karst area (GO)	TS
Pselaphinae indet. 3	Sandstone / Chapada Diamantina region (BA)	TS
*Arthimius* sp.	Limestone / Açungui group / Alto do Ribeira karst area (SP)	F, G, H, L
*Syrbatus* sp. 1	Limestone / Bambuí group / Pains region (MG)	F, H, L
*Syrbatus* sp. 2	Granitic / Serra do Mar / Rio de Janeiro region (RJ)	F, H, L
Tr. Brachyglutini indet.	Limestone / Açungui group / Alto do Ribeira karst area (SP)	F, H
cf. *Strombopsis* sp.	Limestone / Açungui group / Alto do Ribeira karst area (SP)	F, G, H, L
Tenebrionidae indet.	Granitic / Serra do Mar / Rio de Janeiro region (RJ)	F, H, L
Order Hemiptera		
Dipsocoridae indet.	Limestone / Corumbá group / Serra da Bodoquena karst area (MS)	M
Enicocephalidae indet.	Iron ore / Quadrilátero Ferrífero (MG)	L
Ortheziidae indet.	Iron ore / Quadrilátero Ferrífero (MG)	L
Hydrometridae indet.	Limestone / Bambuí group / Serra do Ramalho karst area (BA)	TS
Order Hymenoptera		
Formicidae		
Formicinae indet.	Limestone / Bambuí group / São Desidério karst area (BA)	TS
Ponerinae indet.	Limestone / Bambuí group / São Domingos karst area (GO)	F, G, H, L
Order Orthoptera		
Phalangopsidae indet.	Limestone / Corumbá group / Serra da Bodoquena karst area (MS)	TS
Phylum Mollusca		
Order Caenogastropoda		
Pomatiopsidae		
cf. *Spiropockia* sp.	Limestone / Corumbá group / Serra da Bodoquena karst area (MS)	M
Order Mesogastropoda		
*Potamolithus* sp. 1	Limestone / Açungui group / Alto do Ribeira karst area (SP)	I
*Potamolithus* sp. 2	Limestone / Açungui group / Alto do Ribeira karst area (SP)	I
*Potamolithus* sp. 3	Limestone / Açungui group / Alto do Ribeira karst area (SP)	I
*Potamolithus* sp. 4	Limestone / Açungui group / Alto do Ribeira karst area (SP)	I
*Potamolithus* sp. 5	Limestone / Açungui group / Alto do Ribeira karst area (SP)	D, F, H
*Potamolithus* sp. 6	Limestone / Açungui group / Alto do Ribeira karst area (SP)	I, M.E. Bichuette pers. obs.
cf. *Potamolithus* sp.	Limestone / Corumbá group / Serra da Bodoquena karst area (MS)	L
Order Pulmonata		
Endodontidae indet.	Limestone / Açungui group / Alto do Ribeira karst area (SP)	L
Systrophiidae		
*Happia* sp.	Sandstone / Chapada Diamantina region (BA)	TS
Phylum Chordata		
Order Siluriformes		
Loricariidae		
*Ancistrus* sp.	Limestone / Corumbá group / Serra da Bodoquena karst area (MS)	M
Trichomycteridae		
Trichomycteridae indet.	Limestone / Bambuí group / Pains region (MG)	TS
*Trichomycterus* sp. 1	Limestone / Bambuí group / Serra do Ramalho karst area (BA)	TS
*Trichomycterus* sp. 2	Limestone / Bambuí group / Serra do Ramalho karst area (BA)	TS
*Copionodon* sp.	Sandstone / Chapada Diamantina region (BA)	TS
Heptapteridae		
Heptapteridae indet.	Limestone / Bambuí group / Posse region (GO)	TS
*Rhamdia* sp.	Limestone / Corumbá group / Serra da Bodoquena karst area (MS)	M
*Rhamdiopsis* sp. 1	Limestone / Bambuí group / Cordisburgo region (MG)	E. Trajano pers. comm.
*Rhamdiopsis* sp. 2	Limestone / Una-Irecê group / Chapada Diamantina region (BA)	E. Trajano pers. comm.

In total, eight threats are identified in the Bahia State (Table 3) and the majority of the caves in this State are outside conservation units (natural areas liable for protection by law owing to special features), the exception being in the Andaraí and Lençois regions, where the sandstone caves are recorded inside a conservation unit. For São Paulo State, the amount of threats are fewer (five, Table 3), but there is a concentration of them in areas that contain a high number of subterranean species, e.g., the Alto do Ribeira region – deforestation, land conflicts, pollution of subterranean drainage, small hydroelectric power-stations buildings (SHPS) and uncontrolled tourism.

The most common threat to the hypogean environment (Figure 1, Table 3) was miscellaneous impacts, with historical threats (e.g., deforestation related to agriculture/pastures and mining). For example, from the 29 impacted regions, deforestation for agricultural and/or pastures occurred in 17 (58.6 %); mining in 15 (51.7 %), uncontrolled tourism in six, as is also the case for pollution (20.7 % each); hydroelectric projects are present in five (17.2 %). Roads, land conflicts, gas extraction, and lowering of the water table are more widespread and are present in five regions (17.2 %). Caves included in conservation units are not fully protected - for example, the Açungui group in southeastern Brazil (where there are three State Parks) is under five different threats (Figure 1, Table 3). Specifically, considering the Carajás region in North Brazil, we observed that only mining had an impact that would deplete the entire subterranean environment and lead to the total destruction of landscapes and caves (by mining), with the possible pollution of soil and drainage ways.

**Table 3.** Threats recorded for different Brazilian regions with subterranean taxa. Highlighted in bold, intense degradation activities nowadays; highlighted in italics, potential threats in the near future. SHPS – small hydroelectric power-station buildings.

State / Region	Municipality	Lithology / Geomorphological Unit	Threats
Pará / North Brazil	Altamira region	Sandstone / Altamira-Itaituba group	**Reservoir construction** (Belo Monte) / **Deforestation for pastures**
–	Parauapebas, Curionópolis and Canaã dos Carajas region	Iron ore / Carajás Formation	**Mining**
Mato Grosso do Sul / Central Brazil	Bonito and Jardim regions	Limestone / Corumbá group	**Deforestation for pastures** / Mining projects
Mato Grosso / Central Brazil	Nobres region	Limestone / Araras group	*Hydroelectric project* /*Mining* / *Deforestation for agriculture*
Rio Grande do Norte / Northeastern Brazil	Felipe Guerra and Governador Dix-Spet Rosado regions	Limestone / Apodi group	Mining / Natural gas and oil exploration
Bahia / Northeastern Brazil	Morro do Chapéu region	Limestone / Una-Irecê group	Pollution of subterranean drainages / Deforestation for agriculture / *Mining projects*
–	Iraquara region	Limestone / Una-Irecê group	**Lowering of the water table** / **Uncontrolled tourism**
–	Carinhanha, Coribe, Santana and Santa Maria da Vitória regions	Limestone / Bambuí group - Serra do Ramalho karst area	Deforestation for charcoal production and agriculture / *Mining projects*
–	São Desidério region	Limestone / Bambuí group	*Road construction* (collapses of rock) / *Pollution of subterranean drainage*
–	Itaetê region	Limestone / Una-Irecê group	**Uncontrolled tourism** / **Deforestation for pastures and agriculture**
–	Andaraí and Lençóis regions	Sandstone / Chapada Diamantina	Illegal garimpo / Uncontrolled tourism
	Paripiranga region	Limestone / Canudos supergroup	*Mining projects*
Goiás / Central Brazil	São Domingos region	Limestone / Bambuí group - São Domingos karst area	Uncontrolled tourism / Ilegal mining / Deforestation for pastures and charcoal production
–	Posse and Mambaí regions	Limestone / Bambuí group	Deforestation for pastures, agriculture and charcoal production
	Distrito Federal region	Limestone / Bambuí group	*Mining projects*
Tocantins / Central Brazil	Aurora do Tocantins	Limestone / Bambuí group	Deforestation for pastures and agriculture / *Mining projects*
Minas Gerais / Southeastern Brazil	São Roque de Minas	Limestone / Bambuí group - Serra da Canastra region	Uncontrolled tourism / Deforestation for pastures
–	Jaíba region	Limestone / Bambuí group	**Lowering of the water table** / Pollution of subterranean drainage
–	Presidente Olegário region	Limestone / Bambuí group	**SHPS** / Deforestation for pastures
–	Caeté, Moeda and Brumadinho regions	Iron ore / Quadrilátero Ferrífero	**Mining**
–	Itacarambi and Januária regions	Limestone / Bambuí group	Deforestation for pastures and charcoal production.
–	Cordisburgo region	Limestone / Bambuí group	Uncontrolled tourism (Maquiné cave) / Deforestation for pastures and agriculture
–	Sete Lagoas region	Limestone / Bambuí group	**Mining**
–	Pains region	Limestone / Bambuí group	**Mining**
	Serra da Mantiqueira region	Quartizitic	Deforestation for agriculture / Pollution by pesticides
São Paulo / Southeastern Brazil	Iporanga, Apiaí and Eldorado regions	Limestone / Açungui group - Alto do Ribeira karst area	Uncontrolled tourism / Land conflicts / Pollution of subterranean drainage due to illegal mining and tomatoes plantation / SHPS
–	Itirapina region	Sandstone	Deforestation for pastures and agriculture / Pollution of subterranean darinages
–	Serra do Mar region	Quartizitic	Deforestation for agriculture / Pollution by pesticides
Paraná / South Brazil	Adrianópolis and Rio Branco do Sul regions	Limestone / Açungui group - Alto do Ribeira karst area	SHPS / Deforestation for pastures and agriculture

Considering the described subterranean species up to the end of 2003, only 33 were included in the Brazilian Red List of 2004 and another 30 species were “not reported”. This corresponds to 53 % of the known described subterranean species being included in the IUCN Red List at that time. From 2004 to 2014 we observe augmentation of the Red List, from 33 to 83 species, as well as an increase in the number of described obligatory subterranean species. The majority of these are in the Endangered (EN) or Critically Endangered (CR) categories, compared with the previous Red List, corroborating the fragility of this fauna. Besides there are many species that have not been evaluated (Table 1).

## Discussion

Considering the small number of Brazilian subterranean species recorded to date (150 species plus 156 troglomorphic taxa), we highlight the extreme difficulty in effectively protecting these species. Taxonomic impediment (Linnean shortfall - most of the species have not been described and catalogued (Brown and Lomolino 1998)) is reflected in our results, including specimens of known taxa that have been stored for over 20 years that still are undescribed (e.g., Pseudoscorpiones and Diplopoda). Thus, there is an urgent need for training new taxonomists, since they can accelerate the descriptions, conduct revisionary works, and then include obligatory subterranean species in the IUCN Red List.

As observed in other studies, São Paulo and Bahia States have the highest numbers of obligatory subterranean species, since the São Paulo cave fauna is the best studied in Brazil (Dessen et al. 1980, Trajano 1987, Trajano and Gnaspini-Netto 1991). Regarding the Bahia State, the extended limestone area associated with the current semi-arid climate conditions and the history of past climates has allowed many possibilities for faunistic isolations (Trajano 1995, Trajano et al. 2016). Indeed, it is in this state that we recorded the highest number of obligatory subterranean species occurring also in other kinds of previously neglected lithologies, such as sandstone (Gallão and Bichuette 2015).

Publication of Decree 6640 and the corresponding Normative Instructions (2009, 2017), which classifies caves in terms of relevance degrees, resulted in suppression of Brazilian cave listings. The NIs recommend that subterranean studies for environmental impact assessment reports (for commercial use of the cave/subterranean habitat, such as mining) include two cave sampling campaigns, one in the dry season and one in the rainy season. Highlighting conceptual problems of the NIs, Deharveng et al. (2009) show that even after 110 samplings in European karstic areas, obligatory subterranean species were found. Subterranean fauna inventories may be so inadequate that many species become extinct, before they are discovered and identified (Schneider and Culver 2004, Zagmajster et al. 2014). Thus, adequate sampling methods in different habitats are extremely relevant (Brancelj 2002, Bichuette et al. 2015). Poor subterranean studies represent another problem considering cave conservation. Trajano and Bichuette (2010b) and Trajano et al. (2012) stressed that inadequate sampling designs for evaluation of taxonomic and ecological characteristics leads to biased conclusions, and consequently compromises the conservation of these habitats.

According to Primack and Rodrigues (2001), some species are especially vulnerable to extinction and occur in the following categories: *limited occurrence area*; *one or few known populations*; *small populations*; declining populations; *low population density*; need huge habitats; large species; *species that are not effective dispersers*; seasonal migrants; *low genetic variability*; *species that require special niches*; *species that occur in stable environments*; permanent or temporary aggregations species; and hunting or consumed species. Among these fourteen categories, obligatory subterranean fauna fit at least eight of them (highlighted in italics), revealing the fragility and vulnerability of this fauna.

Although the extent and intensity of deforestation have been relatively high in our study area, reservoir construction for hydroelectric power stations and mining projects are worse threats because these can cause total destruction or irreversible impacts (total removal or flooding) of subterranean habitats, which could lead to fauna extinction as a result of physical destruction of the habitat (Culver 1986). According to Groombridge (1992), habitat loss is the most harmful threat to vertebrates as well as invertebrates, reinforcing the harm caused by the above activities, which can decimate cave fauna.

Recognition of the importance and fragility of subterranean environments by government agencies is becoming apparent with inclusion of obligatory subterranean fauna in threatened species lists. Gallão and Bichuette (2012) stressed the importance of the IUCN Red List for the protection of obligatory subterranean fauna in Brazil. When there is such inclusion, the cave is categorized as ‘maximum totally avoiding cave destruction/suppression’, thus, the IUCN Red List becomes one of the most important tools for protecting caves in Brazil. The IUCN Red List is also an important tool for obligatory subterranean species conservation, since it is one element (among others, see Trajano and Bichuette (2010b) for a review) that includes hypogean habitats as having maximum relevance according to the new Brazilian speleological laws (Decree 6640; see Trajano 2010, 2013, Trajano and Bichuette 2010b). Another relevant and critical point is that, with the inclusion of subterranean species in the IUCN Red List, the whole habitat is being protected. Despite caves with several subterranean species being existing conservation priorities, inclusion of a single subterranean species should be enough to protect the entire cave. However, it is important that we try to protect the entire system, i.e., the cave itself, the surroundings, and the hydrographic basin and/or landscape (Gallão and Bichuette 2012).
